# Extraskeletal Chondroma: Another Diagnostic Possibility for a Soft Tissue Axillary Mass in an Adolescent

**DOI:** 10.1155/2011/309328

**Published:** 2011-12-27

**Authors:** Ibrahim Adaletli, Tal Laor, Hong Yin, Daniel J. Podberesky

**Affiliations:** ^1^Department of Radiology, Cincinnati Children's Hospital Medical Center, 3333 Burnet Avenue, Cincinnati, OH 45229, USA; ^2^Division of Pathology, Cincinnati Children's Hospital Medical Center, OH 45229, USA

## Abstract

Extraskeletal chondroma is a benign cartilaginous tumor that occurs predominantly in the soft tissues near small joints of the hands and feet. There are rare reports of the lesion in other sites, such as the head, neck, trunk, oral cavity, larynx, and pharynx. We present a case of an axillary mass in a 15-year-old girl who underwent MRI examination and resection, with the ultimate diagnosis of an extraskeletal chondroma, in order to expand the differential diagnosis of an axillary soft tissue mass in an adolescent.

## 1. Introduction

Extraskeletal chondroma, also referred to as chondroma of soft parts [1], is a benign soft tissue tumor composed mainly of hyaline cartilage with no connection to bone or periosteum. It is usually small, measuring less than 3 cm in diameter and of similar histology to a juxtacortical or periosteal chondroma, which is located between the bone and periosteum. The most frequent sites of involvement for an extraskeletal chondroma are the hands and feet [2]. Less commonly, it is found in the oral cavity, pharynx, trunk, and knee [3, 4]. Extraskeletal chondroma is a lesion that can be found at any age, but it usually occurs in adults and is very rare in children, with only a few case reports in the literature [4–8].

We present a case of an axillary mass in a 15-year-old girl who underwent magnetic resonance imaging (MRI) examination and resection, with the ultimate diagnosis of an extraskeletal chondroma, in order to expand the differential diagnosis of an axillary soft tissue mass in an adolescent.

## 2. Case Report

**Figure 1:**
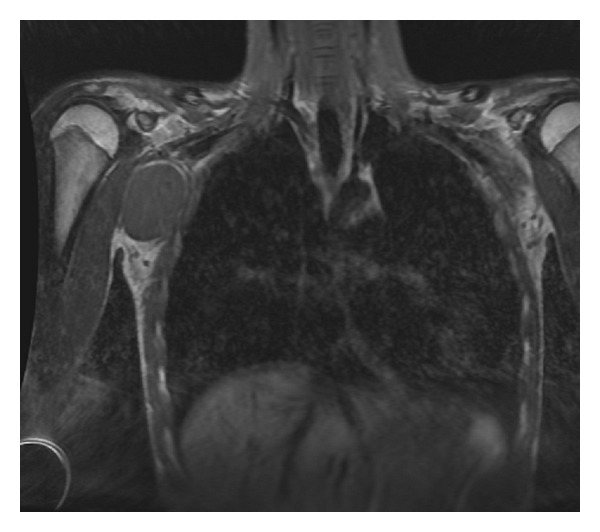
Coronal T1-weighted (500/22 [TR/TE msec]) image of the chest shows a well-defined oval mass of homogeneous signal intensity within the subcutaneous fat of the left axilla.

**Figure 2:**
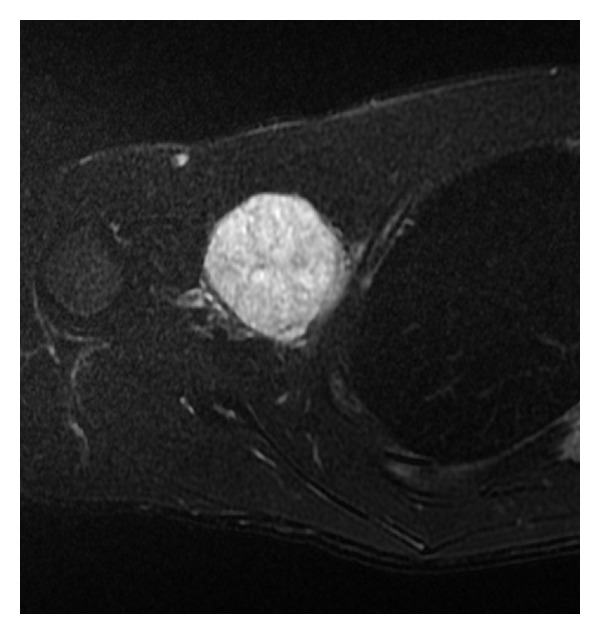
Axial fat-suppressed fast-spin echo T2-weighted (8500/63) image of the right axilla shows homogeneous increased signal intensity throughout the lesion. There is no surrounding soft tissue edema pattern and the adjacent bone marrow signal is normal.

**Figure 3:**
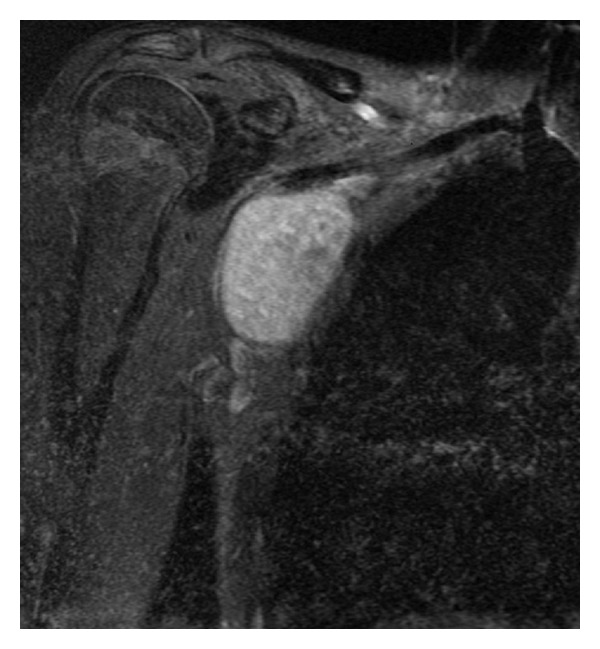
Following intravenous contrast administration, there is homogeneous enhancement throughout the lesion on a fat-suppressed T1-weighted (450/22) coronal image.

An otherwise healthy 15-year-old girl was referred by her clinician to our hospital for imaging of a mass in the right axilla. The mass was slowly growing over the prior 6 months. Physical examination revealed a 5 cm painless, nonmobile, palpable firm lesion in the right axilla. The girl denied tenderness, erythema, or fluctuance. The patient recalls no antecedent traumatic event and has no pertinent medical or surgical history. Her laboratory tests include white blood cell count, erythrocyte sedimentation rate, and C-reactive protein, all of which were normal. Frontal and lateral radiographic views of the chest were normal. MRI was subsequently requested to define the lesion location and to evaluate for any specific imaging characteristics that might propose a diagnosis. MRI showed a well-defined, well-circumscribed, 4 × 5 × 6 cm in diameter solid mass within the subcutaneous fat of the right axilla. The mass was slightly hyperintense to muscle on T1-weighted images (Figure 1) and homogeneously hyperintense to muscle on T2-weighted images (Figure 2). There was marked diffuse homogeneous enhancement throughout the lesion following the administration of intravenous contrast (Figure 3). The remaining subcutaneous fat and adjacent muscle were normal, without surrounding inflammatory changes. Although adjacent to the chest wall, the adjacent ribs were considered normal in morphology and signal intensity. Few normal-sized scattered lymph nodes were present in the axilla, but no lymphadenopathy was noted. The imaging characteristics of the mass were deemed nonspecific, and, because malignancy could not be excluded, tissue sampling was recommended.

**Figure 4:**
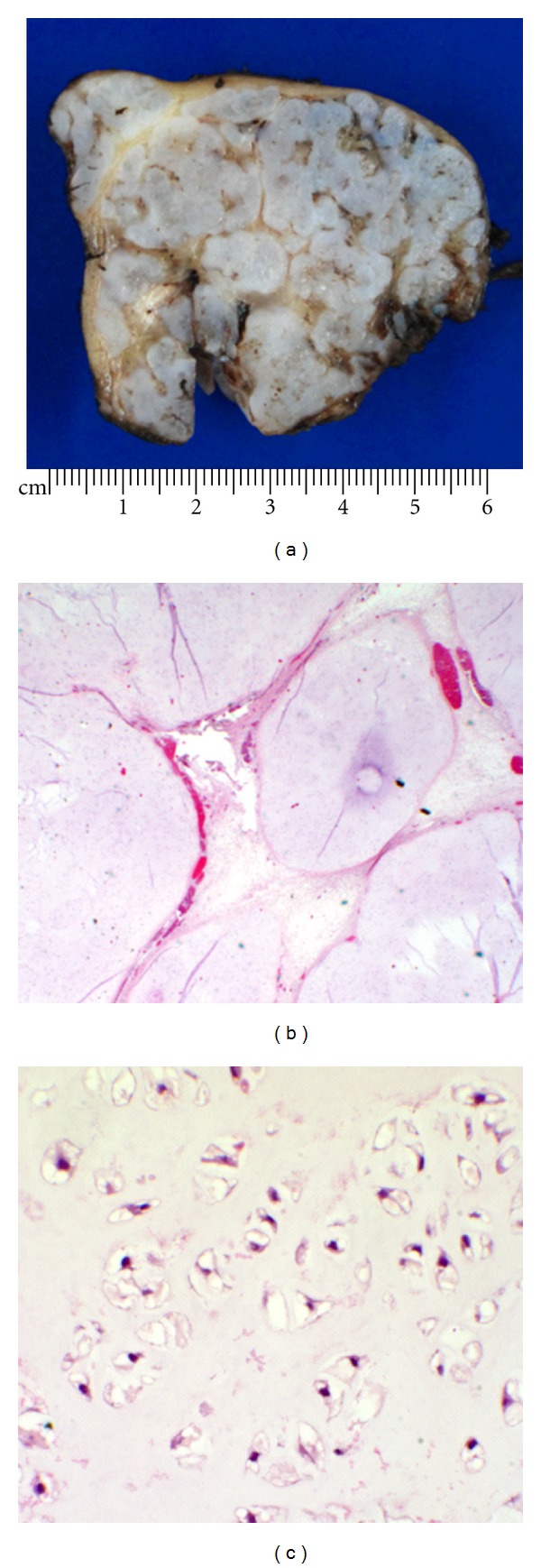
(a) The cut surface of the mass is firm, grayish white, with focal gelatinous areas. (b) Microscopic examination shows islands and elongated lobules of mature benign hyaline cartilage (hematoxylin-eosin, original magnifications ×100). (c) The cartilage consisted of bland appearing chondrocytes in lacunae (hematoxylin-eosin, original magnifications ×400).

Following biopsy and frozen section analysis, the patient underwent full excision of the lesion. The excised mass was well demarcated, multilobulated, measuring 7 × 6.5 × 4.2 cm and weighing 75.9 grams. The cut surface was firm, grayish white, with focal gelatinous areas (Figure 4(a)). Upon microscopic examination, the mass was surrounded entirely by a fibrous capsule. The lesion was composed of islands and elongated lobules of mature benign hyaline cartilage with well-vascularized fibrous stroma (Figure 4(b)). The cartilage consisted of bland appearing chondrocytes in lacunae (Figure 4(c)) with a hypercellular zone at the periphery of the nodules. There was no substantial cytologic atypia, mitosis, or necrosis. No calcification or ossification was present throughout the mass. Histopathologic findings were consistent with an extraskeletal or soft tissue chondroma.

## 3. Discussion

Soft tissue masses of the axilla in children are not uncommon and encompass a wide variety of lesions including both benign and malignant lesions. Most commonly encountered are benign lesions that include enlarged inflammatory or infectious lymph nodes, posttraumatic hematoma, soft tissue abscess, vascular malformation, lipoma, myofibroma, and less commonly accessory breast tissue or fibroadenoma from axillary breast tissue in teens. Schwannoma, myofibroma, and myositis ossificans are additional rare considerations [9]. Malignant lesions include rhabdomyosarcoma, synovial cell sarcoma, and extraskeletal osteosarcoma [1, 9].

 Accessory breast tissue is often asymptomatic, but occasionally it can produce symptoms related to hormonal influences and can be mistaken for a neoplasm [10]. It can be diagnosed by physical examination, ultrasonography, mammography, and/or MRI, with imaging characteristics similar to normal breast tissue. Axillary lymph adenitis usually presents with swelling and tenderness and has typical imaging findings of enlarged, hyperemic masses with a hilar architecture [9]. A lipoma can occur in any location, including the axilla, and is suggested by the fat attenuation on CT or fat signal intensity on MRI. Vascular malformations can be superficial or deep lesions and often have suggestive imaging characteristics on US and MRI [11]. Phleboliths seen on radiography or cross-sectional imaging help to make the diagnosis of a venous malformation. Schwannoma more frequently occurs in the extremities, trunk, or head and is infrequently found in the axilla [12]. The imaging findings of a schwannoma include a well-defined oval homogeneous hypoechoic mass on sonography. On MRI, it is a well-defined mass of intermediate signal intensity on T1-weighted images and of hyperintense signal on T2-weighted images, with diffuse enhancement following intravenous contrast administration, similar to the MRI findings of the extraskeletal chondroma of our report.

 Solitary myofibroma is a single lesion within skin, subcutaneous tissue, muscle, or bone in infant or young children. Myofibromas are frequently confused with both benign and malignant tumors [13]. Myositis ossificans shows a typical zonal pattern peripheral calcification on various imaging modalities but early on this calcification can be inhomogeneous or amorphous [1]. Although calcification is seen in 33–70% of extraskeletal chondromas, the patient in this case report showed neither calcification nor ossification within the lesion on conventional radiography. In light of the lack of mineralization, myositis ossificans, synovial cell sarcoma, and soft tissue osteosarcoma were not considered in the differential diagnosis.

The differential diagnosis from histology includes well-differentiated extraskeletal chondrosarcoma, extraskeletal myxoid chondrosarcoma, and mesenchymal chondrosarcoma. Well-differentiated extraskeletal chondrosarcoma shows abnormal mitoses, atypism, and necrosis. Extraskeletal myxoid chondrosarcoma is less differentiated, especially in the peripheral portion of the tumor. Mesenchymal chondrosarcoma is another chondroid lesion with a characteristic dimorphic pattern composed of well-differentiated cartilage surrounded by small, undifferentiated tumor cells. Our case showed the typical pattern of chondroma without atypia or mitosis and, therefore, was easily diagnosed as a chondroma.

Extraskeletal chondroma is a relatively rare, benign, slow-growing soft tissue tumor that usually occurs in the soft tissues about the joints of the hands and feet [14] and usually measure less than 3 cm in diameter. This tumor is thought to arise from the fibrous stroma of soft tissues, rather than originating from mature cartilaginous or osseous tissue. Extraskeletal chondroma typically affects adults, usually between the ages of 30 and 60 years [15]. It is a very rare lesion, with only a few cases reported in children in the literature [4–8]. The typical clinical presentation of an extraskeletal chondroma is a painless, slowly enlarging nodular soft tissue mass that may be present for a variable amount of time prior to diagnosis. On conventional radiography, an extraskeletal osteochondroma may appear as a well-circumscribed, lobulated mass with dense central mineralization [15, 16]. Calcification is usually ringlike, punctate, or granular, suggesting the presence of hyaline cartilage. Sometimes, mineralization has an unusual configuration or is completely absent, as in our case. CT can confirm the extraskeletal location of the mass and show foci of calcification or ossification that can direct the diagnosis towards extraskeletal chondroma [16]. MRI delineates the lesion location and margins, but the appearance is nonspecific. Extraskeletal chondromas have been described as showing low-to-intermediate signal intensity on T1-weighted images and heterogeneous intermediate-to-hyperintense signal from the cartilages on T2-weighted images [3, 4, 15]. In our case, the mass appeared of slightly hyperintense signal compared to muscle on T1-weighted and hyperintense on T2-weighted sequence. After intravenous contrast administration, marked diffuse contrast enhancement was seen within the mass. Because of the lack of specific imaging characteristics and resultant inability to exclude a malignant tumor, tissue sampling was urged. On pathological evaluation, extraskeletal chondromas are primarily cartilaginous lesions, showing well-encapsulated lobules of mature hyaline cartilage with varying degrees of cellularity. Marginal surgical excision is usually the treatment of choice with preservation of adjacent bone and soft tissue structures.

 To our knowledge this is the first report case of an extraskeletal chondroma of the axilla in an adolescent confirmed by histopathology to undergo MRI evaluation. Only one other case of extraskeletal chondroma located in the axilla has been reported in a child in the literature [6]. When a well-demarcated soft tissue mass with typical central calcifications or areas of ossification is seen on conventional radiography and/or CT, extraskeletal chondroma might be considered in the differential diagnosis. However, a soft tissue mass which displays no characteristic imaging findings should undergo tissue sampling to exclude a malignant soft tissue tumor.
